# Controlled growth of ZnO nanoparticles using ethanolic root extract of Japanese knotweed: photocatalytic and antimicrobial properties†

**DOI:** 10.1039/d2ra04202a

**Published:** 2022-11-01

**Authors:** Miha Ravbar, Ajda Kunčič, Lev Matoh, Sonja Smole Možina, Martin Šala, Andraž Šuligoj

**Affiliations:** University of Ljubljana, Faculty of Chemistry and Chemical Technology Ljubljana Slovenia andraz.suligoj@fkkt.uni-lj.si; University of Ljubljana, Biotechnical Faculty Ljubljana Slovenia; National Institute of Chemistry Ljubljana Slovenia

## Abstract

Synthesis of zinc oxide (ZnO) nanoparticles (NPs) was mediated by plant extracts to assist in the reduction of zinc atoms during the synthesis and act as a capping agent during annealing. The preparation used ethanolic extracts from the roots of Japanese knotweed (*Fallopia japonica*). Two major outcomes could be made. (i) A synergistic effect of multiple polyphenolic components in the extract is needed to achieve the capping effect of crystallite growth during thermal annealing at 450 °C characterized by an exponential growth factor (*n*) of 4.4 compared to *n* = 3 for bare ZnO. (ii) Synergism between the ZnO NPs and plant extracts resulted in superior antimicrobial activity against both Gram-positive bacteria, *e.g.*, *Staphylococcus aureus*, and Gram-negative bacteria, *e.g.*, *Escherichia coli* and *Campylobacter jejuni*. The materials were also tested for their antimicrobial activity against *S. aureus* under ultraviolet (UV) illumination. Also here, the photocatalyst prepared with plant extracts was found to be superior. The residues of the plant extract molecules on the surface of the catalyst were identified as the main cause of the observed differences, as proved by thermal gravimetry. Such a preparation using ethanolic extract of *Fallopia japonica* could serve as a more controlled synthesis of ZnO and potentially other metal oxides, with low environmental impact and high abundance in nature.

## Abstract 2

Synthesis of zinc oxide (ZnO) nanoparticles (NPs) was mediated by plant extracts to assist in the reduction of zinc atoms during the synthesis and act as a capping agent during annealing.

## Graphical abstract

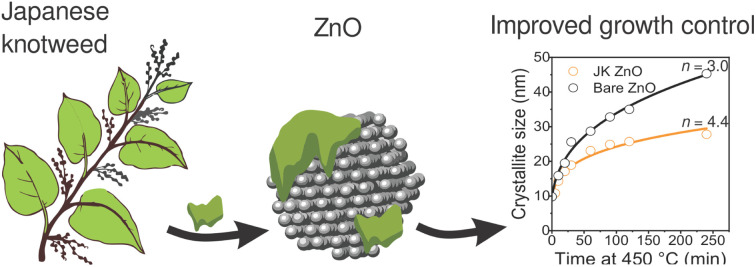

## Introduction

Photocatalysis, a part of advanced oxidation processes (AOP), offers a compelling way of environmental remediation of micropollutants, *i.e.*, recalcitrant pollutants present in the environment in low concentrations. Among many semiconductors capable of exerting the photocatalytic effect, zinc oxide (ZnO) is one of the most appropriate.^1^ This is due to its facile preparation, appropriate electronic band positions, high catalytic efficiency, and relatively good adsorption properties.^2,3^ In fact, the use of ZnO in sun blocking applications may overtake nano-titanium dioxide (TiO_2_) as it can absorb both UV-A and UV-B radiation while TiO_2_ can only block UV-B. This fact can also be beneficial in the photocatalytic sense, as higher absorption of the UV part of the solar spectrum is desired for applications relying on the sunlight irradiation.

The synthesis of metal oxide nanoparticles (NPs) has attracted great interest in recent years. However, to reduce the environmental impact of material preparation, novel synthetic routes need to be found. These can vary from the swapping of precursors/reagents^4^ to the combination with natural materials to obtain the desired properties.^5,6^ For a route to be considered low impact, it must satisfy at least some of the following requirements: (i) it must be made using no or low toxic precursors, (ii) it must consume as low amount of energy as possible, and (iii) it should have high efficiency and produce as little waste as possible. Currently, plant-mediated biological synthesis of NPs is gaining importance due to its simplicity, eco-friendliness and extensive antimicrobial activity.^7,8^

Japanese knotweed (lat. *Fallopia japonica*) is in Europe and America a highly invasive plant, originating from Asia. It invades the habitats of other native species and thus presents a competition for limited resources.^9^ The presence of various phytochemicals in parts of this plant species has great potential for usage in the synthesis of semiconductor NPs.^10,11^ Root extracts of Japanese knotweed have been shown to contain numerous polyphenolic compounds such as epicatechin, resveratrol malonyl hexoside, emodin, catechin *etc.*,^12,13^ which are good candidates as an aid in reduction of Zn atom from acetate precursor. These compounds could also serve as a structure-directing agent for the growth of specific planes and/or geometries during the sintering of the materials.^8^

Although green syntheses of various metal oxide NPs have been widely reported in the literature, most of the reports focus on the synthesis yields or the catalytic/antimicrobial aspects of the material.

Herein, we focus on the impact of the extract of Japanese knotweed on the structural and photocatalytic properties of ZnO NPs. To the best of our knowledge, this is the first study using ethanolic extracts of *Fallopia japonica* to produce ZnO nanoparticles. We pay special attention to the impact on the reusability of ZnO NPs upon multiple cycles of photocatalytic degradation of model pharmaceutical compound ciprofloxacin which is a broad-spectrum fluoroquinolone antimicrobial compound, active against both Gram-positive and Gram-negative bacteria, often used to treat bacterial infections.^14^ It has been found in hospital wastewaters, sewage treatment plants and surface water.^15–18^ Specific conditions can lead to its environmental concentrations from 5 to 20 000-fold higher than reported usually,^19^ while it can also adsorb to sludge, sediment and soil.^20–23^ We show that the use of *Fallopia japonica* extract has beneficial effects both on the structural as well as on the photocatalytic and antimicrobial properties of ZnO NPs.

## Experimental

### Materials

#### Preparation of plant extracts

20 g of dried ground roots of Japanese knotweed was added into a 500 mL Erlenmeyer flask, along with 200 mL of absolute ethanol. Ethyl alcohol was selected based on the high extraction yield reported by others.^13^ The extraction occurred in a covered flask on a magnetic stirrer at room temperature overnight. The mixture was then filtered through 0.45 μm filter paper. A clear solution was collected, and ethanol was added up to the 200 mL mark.

#### Synthesis

A wide 25 mL reaction vial was charged with 220 mg of Zn(CH_3_CO_2_)_2_·2H_2_O (Zn(OAc)_2_, 1 mmol) and 63 mg of LiOH H_2_O (1.5 mmol). 10 mL of ethanol (or ethanol extract of Japanese knotweed) was then added, and the reaction mixture was stirred on a magnetic stirrer at 80 °C for 4 h. After that, the reaction mixture was allowed to cool down to room temperature. The mixture was centrifuged, and the precipitate was washed and centrifuged again, twice with water and one more time with ethanol. The product was air dried before being ground to a fine powder. Additional synthesis reactions were performed by varying the amounts of LiOH H_2_O (0.5–3 mmol), extract amount, time of the reaction (2–24 h) and temperature (RT., 60 °C). One synthesis was also carried out using the above procedure but replacing the extract with a solution of 10 mg of emodin in 10 mL of ethanol.

#### Characterization

Thermal gravimetric analysis (TGA) was performed using a TGA/SDTA 851 apparatus (Mettler Toledo, USA) under nitrogen flow with a heating rate of 10 °C min^−1^. Approximately 20 mg of sample was placed in a platinum crucible on the pan of the microbalance and heated from room temperature to 700 °C, using Al_2_O_3_ as an inert material.

Absorption spectra of the materials were recorded on a Lambda 650 UV-vis spectrophotometer (PerkinElmer, USA), equipped with a Praying Mantis accessory (Harrick). The scan speed was 480 nm min^−1^ and the slit was set to 2 nm. Spectralon® was used for background correction. The photoluminescence (PL) spectra of the ZnO NPs were analyzed using a fluorescence spectrophotometer LS55 from PerkinElmer (USA) with an excitation wavelength of 350 nm at room temperature. For this, the samples were pressed into a disc (*ϕ* = 8.2 mm) under 5 tons of pressure. The attenuated total reflectance FT-IR/ATR spectra were recorded on a Spectrum 100 spectrometer (PerkinElmer, USA) using MIR TGS detector. Spectra were recorded from 4000 to 400 cm^−1^ with the resolution of 2 cm^−1^ using diamond crystal in a horizontal position.

X-ray powder diffraction (XRD) patterns were recorded on a X'Pert PRO (PANalytical, the Netherlands) high-resolution diffractometer using CuKα_1_ radiation (*λ* = 1.5406 Å).

Nitrogen physisorption isotherms were measured on a Tristar 3000 apparatus (Micromeritics, USA) recording at 77 K. The samples were outgassed at 110 °C for 2 h.

Scanning electron microscope SUPRA 35 V P (Carl Zeiss, Germany) field-emission scanning electron microscope operating at 2 kV and using a 30 μm aperture was used to examine the surface morphology of the synthesized catalysts was used to observe the textural features of the materials. Mesoporous characteristics, the presence of manganese oxide species and their structural correlation with mesoporous matrix were investigated by high-resolution transmission electron microscopy (HRTEM) and scanning transmission electron microscopy (STEM). Analysis was carried out on a probe Cs corrected scanning transmission electron microscope ARM 200 CF (Jeol, Japan) with the cold-FEG cathode, equipped with the Dual-EELS system Quantum ER from Gatan and Centurio EDXS system with a 100 mm^2^ SDD detector at 80 kV. For TEM studies a drop of an ethanol diluted sample solution was placed on a nickel grid and dried at room temperature.

LC-MS analysis were performed on UltiMate 3000 UHPLC system (Thermo Scientific, USA) coupled with a triple quadrupole/linear ion trap mass spectrometer (4000 QTRAP LC-MS/MS System; Applied Biosystems/MDS Sciex, Ontario, Canada). Methanol (Chromasolv LC-MS grade, Fluka, Switzerland) and water purified on a Milli-Q system from Millipore (USA) were used for the preparation of mobile phases, formic acid from Fluka was used as modifier. An analytical HPLC column InfinityLab Poroshell 120 EC-C18, 4.6 × 50 mm, 2.7 μm from Agilent was used was used with the flow rate of 0.5 mL min^−1^. A mobile phase consisted of methanol and water, both modified with 0.1% formic acid was used throughout the work. Injection volume and column temperature were 20 μL and 45 °C, respectively. The gradient program was as follows: 0 min: 15% B, 3 min 25% B, 5 min 30% B, 8 min 45% B, 11 min 85% B, 18 min 95% B 26 min 95% B, 26.1 min 15% B and 30 min 15% B. The available standards of polyphenols were emodin (Extrasynthese, analytical standard), polydatin (Merck Sigma, ≥95%) and resveratrol (Sigma, 99%).

#### Activity tests

##### Photocatalytic degradation

The degradation of ciprofloxacin was carried out in a 100 mL crystallizing dish, using a custom made photoreactor.

The reactor consisted of an aluminum chassis equipped with three low-pressure Hg lamps (Actinic BL, 15 W, Philips) and two small fans on the lid as well as a magnetic stirrer, hence providing a top-down height-adjustable illumination. 20 mg of the prepared ZnO powder were weighed into the dish. The dish was equipped with a magnetic stir bar and 40 mL of 10 mg L^−1^ of ciprofloxacin solution was added. The mixture was left to stir in the reactor for 1 h before turning on the lights. At the appropriate time intervals 1 mL aliquots of the reaction mixture were sampled. The mixture was filtered (0.45 μm PA, Sartorius), and a UV-vis spectrum of the clear solution was recorded on a Spectrum 1000 from Varian (USA) with peak wavelength set at 270 nm.

##### Degradation with scavengers

The quenching study of ciprofloxacin was carried out using a similar procedure as described above. Before degradation, scavengers were added into the ciprofloxacin solution. The amount of scavengers added was roughly 20 times that of ciprofloxacin in the solution. *t*-BuOH was added as 24 μL of 1 M solution. Copper(ii) nitrate was added as 120 μL of 0.2 M solution. EDTA was added as 7 mg of solid EDTA. When using D_2_O, a fresh solution of ciprofloxacin was prepared using D_2_O as a solvent instead of H_2_O.

##### Stability testing

The degradation procedure when testing stability was slightly different. 40 mg of ZnO powder was added into a crystallizing dish. Then, 80 mL of 10 mg L^−1^ solution of ciprofloxacin was added and the mixture was stirred for 1 h in the dark to achieve adsorption–desorption equilibrium. The degradation was monitored by using a UV-vis spectrometer with 1 mL sample. After the degradation was complete, the mixture was left under light for two more hours to completely degrade any excess ciprofloxacin. The solution was then dried at 80 °C overnight and the dry ZnO powder was scraped from the bottom of the plate. Fresh 80 mL of the ciprofloxacin solution was added into the dish and the whole procedure was repeated for a total of 10–12 times.

##### Antimicrobial activity

###### Bacterial strains and growth conditions

The bacterial strains *i.e.*, *Staphylococcus aureus* ATCC 25923, *Escherichia coli* ATCC 11229, and *Campylobacter jejuni* NCTC 11168, were provided from the Laboratory for Food Microbiology, Biotechnical Faculty, University of Ljubljana. *S. aureus* and *E. coli* strains were stored at −80 °C in 20% glycerol and 80% Tryptic Soy Broth (TSB, Biolife, Italy) solution, while *C. jejuni* strain was stored at −80 °C in 20% glycerol and 80% Muller-Hinton Broth (MHB, Oxoid, UK) solution. *S. aureus* and *E. coli* were revitalized and further grown on Tryptic Soy Agar (TSA, Biolife, Italy) at 37 °C under aerobic conditions. *C. jejuni* was revitalized by growing on Karmali selective media (Oxoid, UK) at 42 °C for 24 h under microaerophilic conditions (5% O_2_, 10% CO_2_ and 85% N_2_), followed by growing on Muller-Hinton Agar (MHA, Oxoid, UK) at 42 °C for 24 h under microaerophilic conditions.

###### Determination of minimal inhibitory concentrations

The minimal inhibitory concentrations (MICs) of preparations *i.e.*, bare ZnO NPs (ZnO), ZnO NPs synthesized using Japanese knotweed ethanolic extract (ZnO-JK) and Japanese knotweed ethanolic extract (JK), against selected bacteria were determined by broth microdilution method, as previously described^24^ with some modifications. Stock solutions of the material were prepared in sterile distilled water (sdH_2_O) in concentration of 80 mg mL^−1^. The highest concentration of preparations tested was 2 mg mL^−1^ and it was two-fold serial diluted in TSB/MHB to the lowest concentration tested, *i.e.*, 62.5 μg mL^−1^ for testing antimicrobial activity against *S. aureus* and *E. coli*, and about 1 μg mL^−1^ in case of *C. jejuni*. Bacterial inoculum contained approximately 10^5^–10^6^ colony-forming units (CFU) per mL. *S. aureus* and *E. coli* cell viability was determined using INT (2-*p*-iodophenyl-3-*p*-nitrophenyl-5-phenyl tetrazolium chloride, Sigma, USA) solution. *C. jejuni* cell viability was determined using resazurin fluorescent dye solution as previously described,^25^ followed by measuring fluorescence using a microplate reader (Varioskan LUX, Thermo Fisher Scientific, USA) at excitation wavelength 560 nm and emission wavelength 590 nm. MICs were determined at the lowest concentration that inhibited bacterial growth. All experiments were carried out in triplicate.

##### Antimicrobial activity of ZnO nanoparticles after UV irradiation

The manner in which UV irradiation affects the antimicrobial activity of ZnO NPs against *S. aureus* was determined as follows. A bacterial inoculum containing approximately 10^5^–10^6^ CFU mL^−1^, prepared in saline (0.9% NaCl, Merck, Germany), was incubated together with the preparations *i.e.*, ZnO and ZnO-JK NPs, in sub-inhibitory concentrations *i.e.*, 0.25× and 0.125× MIC. The experiment was carried out on a 24-well plate at 37 °C under aerobic conditions with constant shaking at 300 RPM. The bacteria were irradiated with UV light at 365 nm at a distance of 10 cm for 30 min. Cell viability was determined by counting CFU mL^−1^ on agar plates after 24 h incubation at 37 °C under aerobic conditions. CFU mL^−1^ were determined immediately after the end of UV irradiation and 2 h later. UV-irradiated bacteria without added preparations and non-UV-irradiated bacteria were used as controls. The selected controls also proved that UV irradiation at 365 nm does not damage the bacteria. All experiments were carried out in triplicate.

## Results

### Analysis of ethanolic extract

**Fig. 1:**
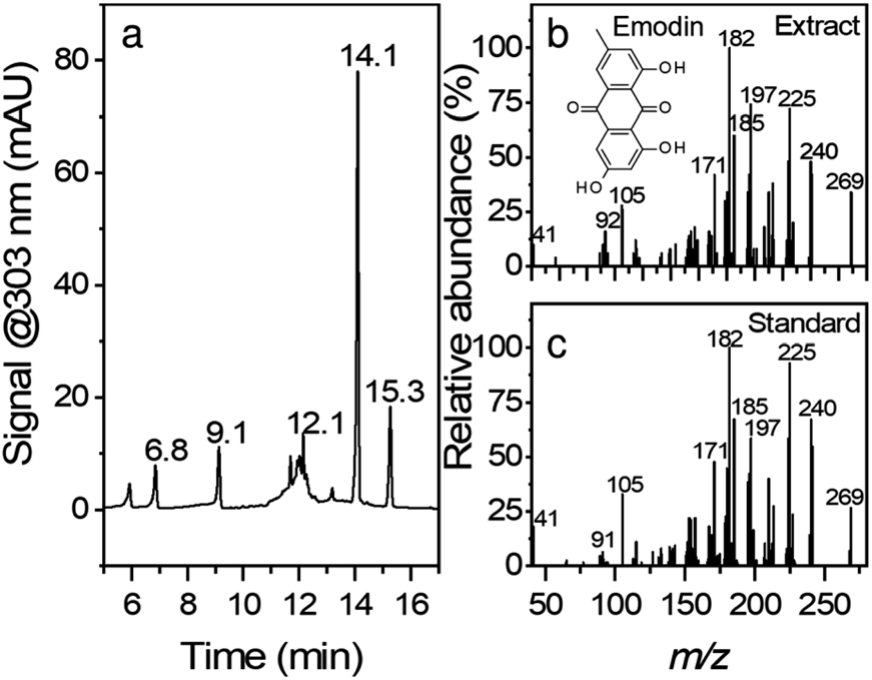
HPLC chromatogram of an ethanolic root extract monitored at 303 nm. MS/MS spectra of peak eluting at 14.1 min in the extract (b) and standard (c). Retention times of the eluted peaks are marked at their respective positions in the chromatogram (a).

Fig. 1 shows the chromatogram and the corresponding MS/MS spectra for peak eluting at 14.1 min. Although root extracts of Japanese knotweed can contain multiple compounds such as stilbenes, anthraquinones, and other phenolic compounds,^26^ the main constituents of the extract in this work were found to be emodin (retention time, RT = 14.1 min), resveratrol (RT = 9.1 min), polydatin (RT = 6.8 min) and emodin glucoside (RT = 12.1 min). The compounds were confirmed with comparison of retention times, UV-vis spectra, and MS/MS fragmentation spectra. In the case of emodin glucoside, where the standard was not available, we have compared the fragmentation spectra of the compound identified as emodin glucoside with the MS/MS spectra of emodin. The fragmentation spectra are nearly identical; hence we conclude that the compound in question is correctly identified as emodin glucoside. Similar example can be seen in the polydatin and resveratrol pair. All the corresponding MS spectra can be found in ESI Fig. S1.†

### Synthesis

The synthesis of ZnO NPs was optimized based on yield. Several parameters were optimized: base/zinc acetate ratio, reaction time, and extract/zinc acetate ratio. However, the base/zinc acetate ratio had the largest influence on the yield of the reaction (Table S1†).

The growth of the ZnO NPs occurs in two stages. In the ethanolic solution, the reactions tentatively proceed as follows.^27,28^ In the first step, Zn(OAc)_2_ is dissolved in ethanol and reduced to form zinc alkoxide.

1 Zn(CH_3_COO)_2_ + 2CH_3_CH_2_OH → Zn(OCH_2_CH_3_)_2_ + 2CH_3_COOH

In the second step, the addition of LiOH gives rise to the zinc condensation process to produce a ZnO precipitate.

2 Zn(OCH_2_CH_3_)_2_ + 2CH_3_COOH + 2LiOH →ZnO↓ + 2(CH_3_COO)Li + 2C_2_H_5_OH + H_2_O

These reactions represent the first stage, that is, the directional attachment and bonding mechanism. The second stage is the maturation, where smaller ZnO particles grow when energy is provided in the form of an annealing temperature. The growth process can be explained by Ostwald's ripening phenomenon, which is a thermodynamically driven process of dissolution of small crystals or sol particles and the redeposition of the dissolved species on the surfaces of larger crystals or sol particles. It has been shown that for ZnO this phenomenon occurs through oriented attachment.^29^

The residues of lithium acetate and ethanol are discarded and precipitate of ZnO NPs is collected. Although the compounds in the extract could aid the reduction of zinc alkoxide, the effect seems to be of minor importance. Low amounts of LiOH resulted in very small yields, while amounts larger than 1.5 mmol resulted in yields greater than 100%. This implies that (i) LiOH is instrumental for reduction of Zn alkoxide, and (ii) side products were also precipitated and isolated during the synthesis. It has been shown that the amount of acetate groups on ZnO NPs depends on the ratio of LiOH : Zn(OAc)_2_.^30^ Thus, the increased yields at higher LiOH amounts are a consequence of COO^−^ groups on the surface of NPs that reacted with polyphenolic compounds in *Fallopia japonica* extract giving the extra mass. Therefore, 1.5 mmol was determined to be the optimal amount of base with a yield of 83%.

### Characterization

**Fig. 2:**
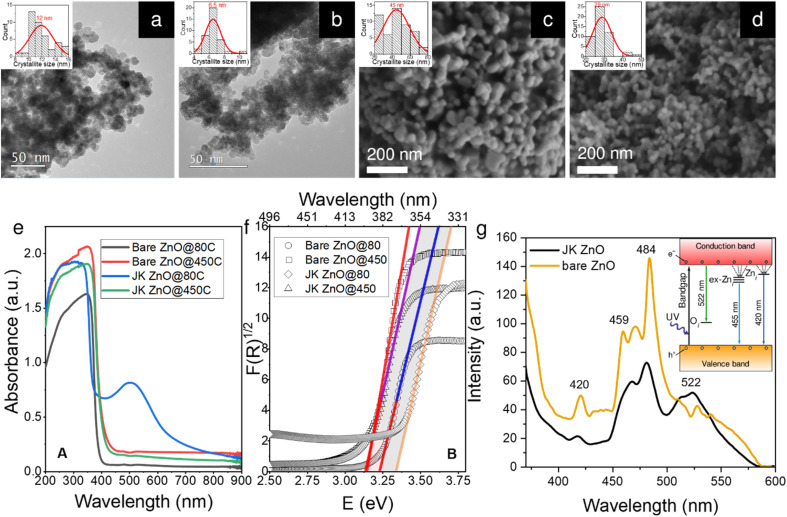
Characterization results of ZnO nanoparticles. TEM micrographs of ZnO NPs synthesized at low temperature (80 °C); bare ZnO (a) and JK-ZnO (b), and SEM images of ZnO NPs after calcination at 450 °C for 4 h; bare ZnO (c), JK-ZnO (d). The insets show a normal distribution of NP sizes. UV-vis diffuse reflectance spectra (e) and their corresponding Tauc plots (f) of NPs prior and post thermal treatment, and (g) PL spectra of the NPs treated at 450 °C.

Well-resolved ZnO crystallites can be seen in the TEM and SEM microphotographs (Fig. 2a–d), confirmed also by selected area electron diffraction (SAED) patterns (Fig. S2†). However, the sizes differ slightly depending on the synthesis method. While the ZnO NPs synthesized without the use of the extract have a diameter of approximately 12 nm, those prepared using the extract are slightly smaller, *i.e.*, approximately 7 nm. We will discuss this observation later in the context of the XRD results. The size distribution of the two samples also differs slightly. As shown by the insets in Fig. 1a–d, the sample prepared using the plant extract has a narrower unimodal size distribution, while the sample prepared without the extract has a much broader distribution with a considerable fraction of particles larger than 14 nm in size.

These differences persisted after calcination at 450 °C for 4 h (Fig. 2c and d). Note that this time is more than sufficient to achieve effective crystallinity and is shown here to reveal the differences between the two samples. A more in-depth study of the effects of annealing time on the crystallinity of the samples will be shown later in the XRD study. Here, the images from SEM show that the size is again significantly smaller in the case of the plant extracts (29 *vs.* 44 nm). This is also reflected in the size distribution, where the sample with the plant extract has again a narrower unimodal distribution, which contrasts with the broader distribution of the pure ZnO sample.

The absorption spectra of the powder samples (Fig. 2e) show that the materials absorb at the edge of the visible range at around 400 nm. When calcined at 450 °C for 10 min, a clear trend can be seen. Both bare ZnO and ZnO prepared with plant extracts show a red shift of the absorption onset to about 420 nm. This can be attributed to the enlargement of the NPs during calcination (see below). When the results are converted according to the Kubelka–Munk theory (Fig. 1f),^31^ the energy of the absorption band edge is obtained. The bandgap energy (*E*_g_) is the largest for low-temperature ZnO using the Japanese knotweed extract, while it is lower by about 0.2 eV for the sample without extract. When the samples were heated, *E*_g_s were lowered because of the increase in the crystallite size; here, both samples show similar values of *E*_g_ of about 3.15 eV.

The photoluminescence (PL) spectra of the two samples calcined at 450 °C for 10 min (Fig. 1g) show a strong UV emission peak related to a near-band edge emission of the ZnO crystal and might be attributed to the recombination of free excitons by the exciton–exciton collision process ZnO. The broad emission in the visible range (450–580 nm with peaks at 459, 470, and 484 nm) originates from transition of a photogenerated electron from the conduction band edge to a trap level of various intrinsic defects such as oxygen vacancies (V_O_), oxygen interstitials (O_i_), oxygen antisites (O_Zn_), zinc vacancies (V_Zn_) and zinc interstitials (Zn_i_).^32,33^ The energy diagram is shown in the inset in Fig. 2g. The relatively low intensity of the green emission at 522 nm may be due to the low density of V_O_ during the preparation of the ZnO powders. The differences in both spectra indicate a lower presence of defects in the ZnO prepared with the extract, which can be seen from the lower intensity of the PL absorption bands mentioned above.

**Fig. 3:**
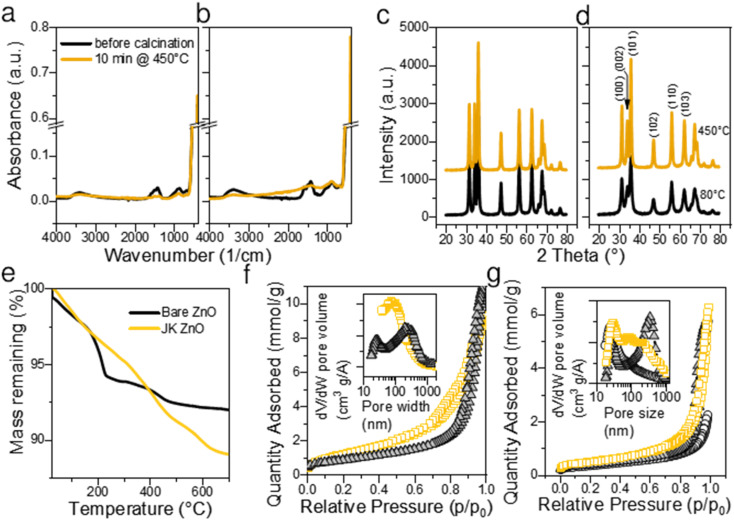
Structural and chemical properties of the prepared NPs. ATR-FTIR spectra before and after calcination at 450 °C for bare ZnO (a) and JK ZnO (b). XRD diffractograms before and after calcination at 450 °C for bare ZnO (c) and JK ZnO (d). The thermogravimetric analysis (TGA) of the synthesized ZnO NPs with and without the extract of *Fallopia japonica* (e) and N_2_ isotherms for samples as synthesized at 80 °C (f) and thermally treated at 450 °C for 10 min (g). The colors in (f and g) are: orange – JK ZnO, grey – bare ZnO, and white – reference ZnO NPs from Aldrich.

The thermal gravimetric analysis of both samples did not show the presence of large amounts of surface water (main loss in the temperature range 30–100 °C). This is a consequence of heat treatment at 450 °C for 10 min. A separate experiment showed that 450 °C is sufficient to complete the calcination. On the TGA curve (Fig. 3e) the main loss of mass was observed between 200 and 250 °C with a maximum at about 210 °C. Weight loss in this range is considered due to the degradation of acetate groups bound to the surface of ZnO NPs, for example, Zn(OAc)_2_ fully decomposes up to 280 °C.^34^ The position of the largest drop at 218 °C indicates a high Zn(OAc)_2_ : LiOH ratio.^30^ However, in our case, the molar ratio Zn(OAc)_2_ : LiOH was 1 : 1.5, which is lower than the values reported by Sakohara *et al.*^30^ The fact that the mass loss was very low suggests low number of additional byproducts such as lithium acetate was formed and that very few unreacted Zn(OAc)_2_ is present on the surface of NPs. On the other hand, JK-ZnO exhibited slightly larger mass loss, and the major step was shifted from 218 °C to 410 °C. As a reference, the ethanolic extract of the root of Japanese knotweed shows the major step at 313 °C. The shape of the TG curve implies lesser number of intermediaries were present during the thermal decomposition in the case of JK ZnO.

FTIR spectroscopy revealed that the ZnO NPs (Fig. 3a) have a pronounced peak at ∼470 cm^−1^ before calcination. The peak is the signature of polar stretching vibrational modes of hexagonal ZnO.^28^ The broad absorption at 3360 cm^−1^ results from the OH stretching vibration,^28^ while the absorption bands at 1564 and 1424 cm^−1^ are the result of the COO^−^ asymmetric and symmetric stretching vibrations, respectively. Out-of-plane C–H bending vibrations are observed at 860 cm^−1^.^7^ The COO^−^ bands are overlaid by a broad band centered at about 1500 cm^−1^ showing the vibrations of ZnO like adsorbed X–OH.^35^ All bands are of similar intensity in both samples, only the bands not belonging to ZnO are slightly more pronounced in the JK ZnO sample. After sintering NPs at 450 °C for 10 min, the Zn–O absorption becomes more pronounced, while at the same time the bands at 3362, 1500, and 900 cm^−1^ disappear. This confirms that thermal treatment serves to remove the residues of adsorbed water and carboxyl compounds from the surface of the catalysts. It also corroborates with the improved crystallinity after sintering.

XRD diffractograms (Fig. 3c and d) reveal that ZnO NPs are crystalline without the presence of an amorphous phase even in low temperature synthesis, that is, before calcination. Indexing revealed that hexagonal wurtzite (JCDPS card no. 01-075-0576) with the space group *P*6_3_*mc* is the only phase in the samples, with preferred orientation along the (101) plane. In particular, the orientation (002) at 34.49° is less prominent in JK ZnO. This orientation has been reported to depend on the annealing temperature, especially in thin ZnO films.^36^ After calcination, the NPs grow. The sizes of the JK ZnO crystallites were 10 and 14 nm before and after calcination at 450 °C for 10 min, respectively, calculated with the Scherrer equation. The orientation (002) is still less prominent in the JK ZnO sample even after heat treatment.

The textural properties determined with nitrogen sorption at 77 K are shown in Fig. 3f and g. All samples show a type II isotherm implying that the adsorption–desorption behavior of N_2_ is a consequence of mesopores. Indeed, the hysteresis loops of type H3 indicate at the interparticle porosity of non-rigid aggregates of plate-like particles. It could also indicate at the pore network consisting of macropores which are not fully filled with pore condensate.^37^ According to the SEM and TEM analysis, both options seem plausible. The pore sizes according to the BJH theory differed between the samples (Table S2†); JK ZnO showed smaller pore sizes compared to bare ZnO both in synthesized and thermally treated samples. Annealing caused an increase in pore sizes in both samples which is due to growth of crystallites. In concurrence, specific surface areas according to the BET theory decreased upon annealing; more notably for JK ZnO, yet still displaying 37% higher areas than those for bare ZnO.

**Fig. 4:**
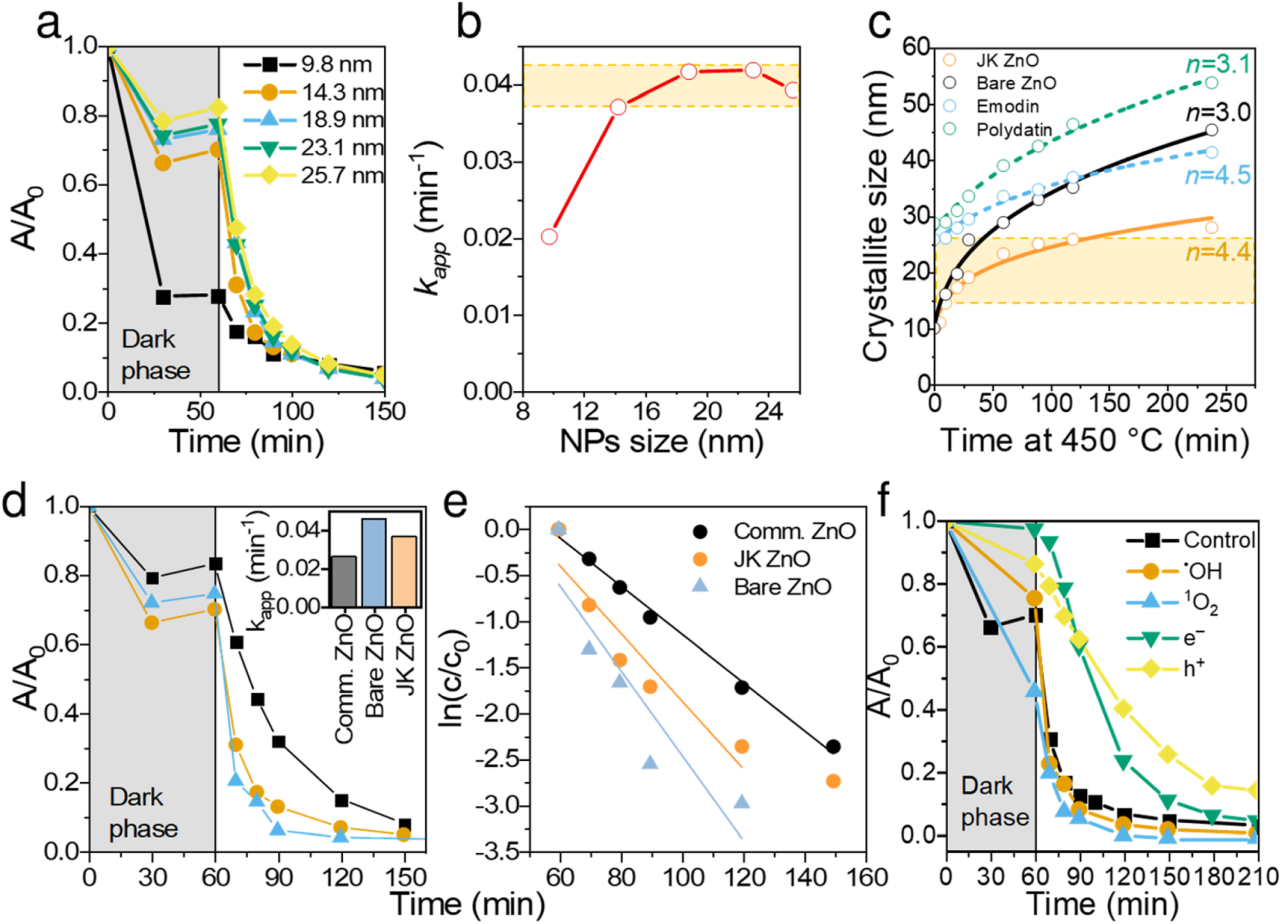
Catalytic and size dependent properties of the prepared ZnO NPs. Degradation kinetics of ciprofloxacin for different-sized NPs (a) and the calculated corresponding apparent reaction rate constants, *k*_app_ (b). Particle size growth when annealing at 450 °C (c) using different extracts or components of extracts; the solid lines represent a non-linear square fit to equation *r*^*n*^ − *r*_0_^*n*^ = *Kt*. Comparison of degradation kinetic behaviors of commercial ZnO, bare ZnO and JK-ZnO nanoparticles (d and e); inset in (d) shows *k*_app_ of the three catalysts. Comparison of CIP degradation kinetics with the addition of scavengers (f).

Continuing the discussion on synthesis, the effect of sintering time at 450 °C was studied for both materials and is shown in Fig. 4c. The presence of extracts of Japanese knotweed decreased the ZnO particle growth rate, thus offering better control over crystallization. The growth kinetics of isothermally sintered ZnO NPs can be described with a parabolic kinetic equation:^38,39^

3 *r*^*n*^ − *r*_0_^*n*^ = *Kt*

where *t* is the annealing time, *r* is the grain size after the annealing time, *n* is the growth exponent, *r*_0_ is the starting grain size and *K* is a temperature-dependent parameter. The later can be expressed in an Arrhenius-type equation:

4 *K* = *K*_0_ exp(−*E*_a_/*kT*)

where *E*_a_ is the activation energy and *k* is the Boltzmann constant.

Generally, the growth of nanocrystals in the absence of any capping agent should yield an exponent (*n*) between 2 and 3; 2 being the limiting value where growth is entirely controlled by surface reaction and 3 being the limiting value when growth is solely limited by diffusion.^39^ In the case of bare ZnO (Fig. 4c), *n* = 3 suggests that thermal growth is limited by diffusion. In the case of ZnO prepared with plant extract, *n* = 4.4, which is considerably higher than that of uncapped ZnO. This suggests that the growth of ZnO nanocrystals is much slower than the usual and expected rate of growth, thus confirming the capping role of plant extracts on the surface of ZnO. It is also evident that synergism of multiple components in the extract is responsible for capping activity. Although emodin showed similar capping activity (*n* = 4.5), polydatin did not show any growth delaying effects (*n* = 3.1).

Thus, ZnO NPs were synthesized and cured with the aid of Japanese knotweed for 10 min for the following reasons: (i) to keep the particle size low but still obtain sufficient crystallization degree, (ii) to obtain NPs with comparable light absorption properties (see Fig. 2e), and (iii) to have NP sizes comparable to bare ZnO, synthesized under the same conditions (see Fig. 4c).

### Photocatalytic activity

The prepared ZnO NPs were investigated in the photocatalytic degradation of ciprofloxacin (CIP), a model antibiotic for cardiovascular drugs, often found in high concentrations in the vicinity of pharmaceutical production facilities.^40^ The degradation was carried out under a UV light (*λ*_max_ = 365 nm) and the concentration of CIP was monitored with a UV-vis spectrometer centered at 271 nm. It should be noted that *λ*_max_ of CIP shifted from 272 to 265 nm from the beginning to the end of degradation, indicating on the disruption of the chromophoric conjugation system. Photodegradation of fluoroquinolone agents is likely to occur at the piperazine ring, F atom, carboxyl group, and cyclopropyl group.^41^ The observed shift agrees with the literature; however, an in-depth analysis of the degradation pathway would be beyond the scope of this paper.

Larger NPs sizes resulted in faster photocatalytic degradation (Fig. 4a and b), which is a consequence of higher crystallinity and thus better light absorption. The apparent reaction rate constant (*k*_app_) increased from 0.02 min^−1^ to 0.038 min^−1^ for 10 and 14 nm NPs, respectively. Further increasing the sizes of NPs increased the *k*_app_ for additional ∼10%, reaching the plateau at ∼18 nm, albeit at the expense of lower adsorption. Thus, the optimal NP size was found in the range 14. Note that NPs of all sizes degraded CIP to completion within 100 min of illumination.

To put these results in perspective, we compared JK ZnO with commercially acquired Aldrich ZnO NP, as well as bare ZnO (Fig. 4d and e). Both JK ZnO and bare ZnO showed higher photocatalytic activities which lead to lower degradation times than the commercially available ZnO NPs. Note that the bare ZnO and JK ZnO showed similar kinetics with slightly better adsorption properties seen with JK ZnO. The latter can be attributed to the larger *S*_BET_ area. These results show the appealing photocatalytic properties of materials for which size and crystallinity can be finely tuned.

To determine the dominant active species responsible for the photocatalytic degradation process with JK ZnO, we carried out several degradations with different scavengers in amounts ten times greater than CIP to ensure sufficient effect (Fig. 4f). The scavengers used were: *tert*-butanol, EDTA, copper(ii) nitrate and D_2_O. *tert*-Butanol is used as a scavenger for hydroxyl radicals (˙OH); hence, if the JK ZnO NP relied on hydroxy radicals for the degradation of organic compounds, the degradation rate should decrease significantly with the addition of *t*-BuOH.^42^ However, here the degradation was comparable when *t*-BuOH was added, meaning that ˙OH is not the dominant species responsible for the degradation. Copper(ii) nitrate can be used as an electron scavenger.^43^ When added to the solution of ciprofloxacin, copper nitrate not only decreased the adsorption of CIP but also significantly decreased the degradation rate when using JK ZnO NPs. This indicates a substantial contribution of free e^−^ to the degradation mechanism of CIP. Ethylenediaminetetraacetic acid (EDTA) acts as a hole (h^+^) scavenger and can therefore slow down the photocatalytic degradation if holes are a major contributor to the degradation mechanism. Here, the effect was highly significant, and EDTA had the largest effect of all the scavengers which shows h^+^ play a major part in the degradation mechanism when using JK ZnO. Finally, D_2_O was used to determine the role of singlet oxygen (^1^O_2_) in degradation. Since the lifetime of ^1^O_2_ is 55 μs in D_2_O and 3.3 μs in H_2_O,^44^ singlet oxygen-mediated reactions should be accelerated in D_2_O compared to H_2_O, due to the longer lifetime of ^1^O_2_ and hence the higher number of active species. In our case, the degradation was only slightly faster compared to H_2_O, indicating that ^1^O_2_ does not contribute significantly to the overall degradation mechanism. Most of these findings are in accordance with a similar study by Makama *et al.*,^45^ who reported that hydroxyl radicals have a marginal role in the degradation mechanism, while valence band holes (h^+^) contributed the most to the degradation of ciprofloxacin. The main difference is that they found free electrons (e^−^) to have a minor to no effect on degradation (Fig. 6). However, this difference could be due to the different type of photocatalyst used, where they used a ZnO/SnS_2_ nanocomposite, while our photocatalyst was pure ZnO nanoparticles. This agrees with research done by Chankhanittha *et al.*,^46^ who found e^−^ to be instrumental for the degradation of ofloxacin. The degradation of CIP could thus be written as:

5 ZnO + *hν* → ZnO + e^−^ + h^+^

6 e^−^ + O_2_ → ˙O_2_^−^

7 ˙O_2_^−^ + H^+^ → ˙HO_2_

8 2e^−^ + ˙HO_2_ + H^+^ → ˙OH + OH^−^

9 e^−^ + CIP antibiotic → degradation products

10 h^+^ + CIP antibiotic → degraded products

The role of e^−^ and h^+^ are thus instrumental to produce reactive oxygen species (eqn (5)–(8)) as well as by performing direct oxidation of CIP (eqn (9) and (10)).

### Evaluation of the photocatalytic stability

To assess the stability and longevity of the prepared ZnO NPs, we carried out 12 consecutive degradations of CIP using the same catalyst. After each completed photodegradation, the mixture of the ZnO catalyst and the remaining solution (now free from CIP) was dried overnight at 80 °C. Fresh solution of CIP was then added onto the dry ZnO and the experiment was repeated for a total of 12 times.

**Fig. 5:**
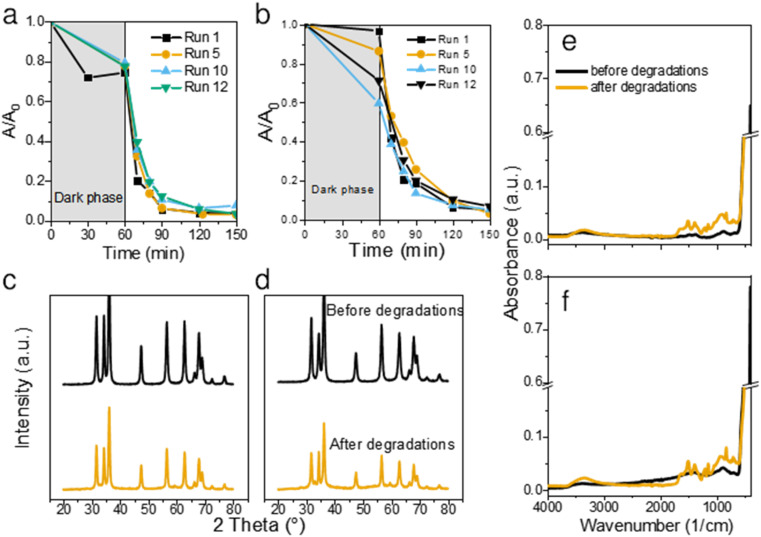
Long-term use of the two catalysts. Kinetics of CIP degradation over 12 consecutive degradation runs using bare ZnO (a) and JK ZnO (b) NPs. Comparison of XRD diffraction patterns before and after 12 subsequent degradation runs for bare ZnO (c) and JK ZnO (d), and FTIR spectra of NPs before and after degradations for bare ZnO (e) and JK ZnO (f).

Both bare ZnO and JK ZnO retained most of their degradation activity after 12 cycles (Fig. 5a and b). With JK ZnO, a steady increase in the adsorption of CIP was observed in subsequent cycles. This could be attributed to the slow removal of leftover organic species on the surface of the nanoparticles after the synthesis. This agrees with the TGA analysis and DRS and FTIR, which indicate the presence of additional surface species in the JK ZnO sample. With bare ZnO, an opposite effect was seen, with a slight decrease in the dark adsorption of CIP. The rate of degradation was stable in both materials; yet a slight gradual decrease in kinetics was observed in bare ZnO with higher cycle numbers, whereas the same effect was not observed in JK ZnO. Despite this, both materials show good long-term stability.

**Fig. 6:**
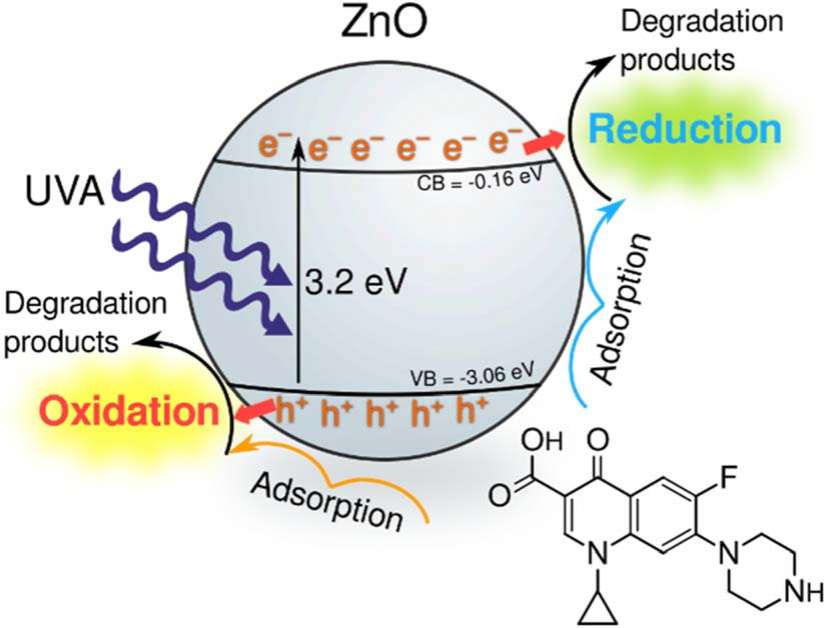
Tentative photocatalytic degradation pathway of ciprofloxacin using the synthesised ZnO NPs under UV irradiation.

**Fig. 7:**
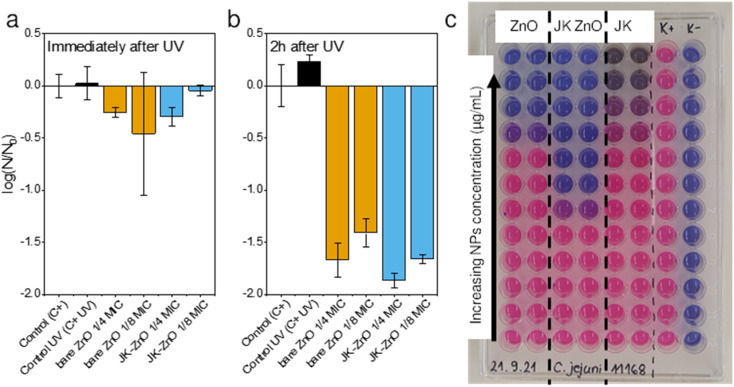
Antimicrobial activity of ZnO nanoparticles immediately after (a) and 2 h after UV irradiation (b) against *S. aureus* (*N* = CFU mL^−1^). Black colors represent controls, orange bare ZnO and blue JK-ZnO. Initial *S. aureus* inoculum (*N*_0_) = 10^6^ CFU mL^−1^. Determination of minimal inhibitory concentration (MIC) for *C. jejuni* (c), expressed in relative fluorescence units (RFU). MIC is the concentration of solids when fluorescence reaches that of the negative control (K−).

To check the stability of the structure, XRD diffractograms were recorded with samples after 12 photocatalytic cycles. A slight decrease in the crystallinity of both materials was observed both in the XRD patterns (Fig. 5c and d) and FTIR spectra (Fig. 5e and f). The diffractograms show a slight decrease in size of NPs, while the FTIR indicate on slightly lower crystallization degree based on the decreased 450 cm^−1^ peak intensity. Nevertheless, neither of these results indicate on a severe drop in structural features. We also note, that in contrast to our previous report,^1^ no trace of lithium acetate was detected in the spent powder, despite the prolonged cycling of the material. Thus, it shows the promising long-term stability of the synthesized photocatalysts. Antimicrobial activity was checked to see whether synthesis with added extracts and growth control affected these properties. In particular, polyphenolic compounds in the ethanolic extract of *Fallopia japonica* should exhibit antimicrobial properties themselves.^13^ We thus included a dried extract powder as an antimicrobial agent as well (Fig. 7).

### Antimicrobial activity

The antimicrobial activity of ZnO NPs and the Japanese knotweed ethanolic extract was determined against Gram-positive bacteria *i.e.*, *Staphylococcus aureus* and two Gram-negative bacteria *i.e.*, *Escherichia coli* and *Campylobacter jejuni* (Table 1). The broth microdilution method was used, as it appeared to be more sensitive than the screening agar methods, *e.g.*, the agar diffusion method, according to our previous studies.^24^ The results showed that ZnO NP synthesized using the Japanese knotweed ethanolic extract exhibited greater antimicrobial activity against selected bacteria compared to bare ZnO NP and the Japanese knotweed ethanolic extract alone (Fig. 7c). For instance, the MIC of JK ZnO NPs against *S. aureus* was four times lower than the MIC of bare ZnO NPs, and eight times lower in case of *C. jejuni*. Only against *S. aureus* the MIC of JK was comparable to that of ZnO-JK NPs.

**Table 1:** Antimicrobial activity of the materials against selected bacteria, expressed as minimal inhibitory concentrations (MICs) in mg L^−1^ determined by broth microdilution method

Sample	MIC (mg L^−1^)		
	*Staphylococcus aureus* ATCC 25923	*Escherichia coli* ATCC 11229	*Campylobacter jejuni* NCTC 11168
ZnO	2000	>2000	500
JK-ZnO	500	2000	62.5
JK	500	>2000	1000

To test a more practical application in which the NPs could be immobilized in a film or brought into contact with a particulate material, we assessed their antimicrobial properties after 30 minutes of UV illumination (Fig. 7a). The antimicrobial activity of ZnO NPs after UV irradiation was determined against the Gram-positive bacteria *S. aureus*. Subinhibitory concentrations of ZnO NPs were tested, *i.e.*, 0.25× and 0.125× MIC, because these concentrations had not affected bacterial growth in the previous experiment. We determined the CFU mL^−1^ immediately after the 30 minute UV irradiation and 2 hours afterward (Fig. 7b). The cell-damaging effect of ZnO-NP was observed 2 hours after UV irradiation, but not immediately thereafter. Additionally, because the MIC of JK ZnO NPs was lower (500 mg L^−1^) than the MIC of bare ZnO NPs (2000 mg L^−1^), the concentrations tested were lower, *i.e.*, 125 mg L^−1^ and 62.5 mg L^−1^ for JK ZnO NPs and 500 mg L^−1^ and 250 mg L^−1^ for bare ZnO NPs. From this, it is concluded that the JK ZnO NPs have a higher antimicrobial activity because the microbial population was reduced by 99% compared to the controls. The experiment also shows that UV irradiation of ZnO NPs increases their antimicrobial activity, which is *in lieu* with the results of the photocatalytic part. Thus, the antimicrobial activity of the JK ZnO NPs was superior to the bare ZnO NPs where 1/8 of MIC reduced the 1.78 log(CFU_0_), compared to 1.59 log(CFU_0_) reduction for bare ZnO.

## Conclusions

Nano-sized ZnO nanoparticles were synthesized *via* a green synthesis method with the aid of a plant extract from the roots of Japanese knotweed (*Fallopia japonica*). Emodin, resveratrol, polydatin and emodin glucoside were detected in the extract. The highest photocatalytic degradation of ciprofloxacin was observed with ZnO NP sizes in the range 15–27 nm. This size range was most easily obtained with synthesis with that used the root extract. However, combination of all components in the extract was needed to achieve the effect of delayed growth of ZnO NPs with thermal treatment at 450 °C. Additionally, synergy between the extract and ZnO NPs was needed for optimal antimicrobial activity against Gram negative as well as Gram positive bacteria. This indicates a potential environmentally friendly usage of this otherwise invasive (at least in Europe and North America) plant species.

## Conflicts of interest

There are no conflicts to declare.

## Supplementary Material

RA-012-D2RA04202A-s001
