# Supplementary material for: Controlled growth of ZnO nanoparticles using ethanolic root extract of Japanese knotweed: photocatalytic and antimicrobial properties
Source: RSC Adv. 2022 Nov 1;12(48):31235–45. doi: 10.1039/d2ra04202a (PMC9623611; doi:10.1039/d2ra04202a)
Supplement: RA-012-D2RA04202A-s001 [file RA-012-D2RA04202A-s001.pdf]

Electronic Supplementary Material (ESI) for RSC Advances.
This journal is © The Royal Society of Chemistry 2022

Supplementary information on the manuscript: Plant extracts-mediated growth of ZnO nanoparticles using Japanese knotweed: Photocatalytic and antimicrobial properties

Miha Ravbar[a], Ajda Kunčič[b], Lev Matoh[a], Sonja Smole Možina[b] Martin Šala[c] and Andraž Šuligoj[a,c,*]

[a] University of Ljubljana, Faculty of Chemistry and Chemical Technology, Department of Inorganic Chemistry, Večna pot 113, SI-1000 Ljubljana, Slovenia

[b] University of Ljubljana, Biotechnical Faculty, Department of Food Science and Technology, Jamnikarjeva 101, SI-1000 Ljubljana, Slovenia

[c] National Institute of Chemistry, Hajdrihova 19, SI-1000 Ljubljana, Slovenia

HPLC determined components in the ethanolic extract are shown below. Standards were injected at concentration of 0.01 mg/mL. Their presence was confirmed with comparing their retention times, their UV-vis spectra and their MS/MS spectra. In the case of emodin glucoside where standard was not available, the structure was deducted based on the MS/MS fragmentation which was nearly identical to fragmentation of emodin. The analogous picture was seen in polydatin–resveratrol pair which also differ by one glucose molecule. Hence, it was reasonable to assignate the peak at 12.1 min to emodin glucoside. We also note the UV-vis spectra were identical, due to identical chromophore from the emodin conjugation system present in both molecules. We were unable to assign the compound eluting at 15.3 by its MS/MS spectrum. Due to its UV-vis being nearly identical to the one of emodin and eluting slightly later we assume it is one of its derivatives.

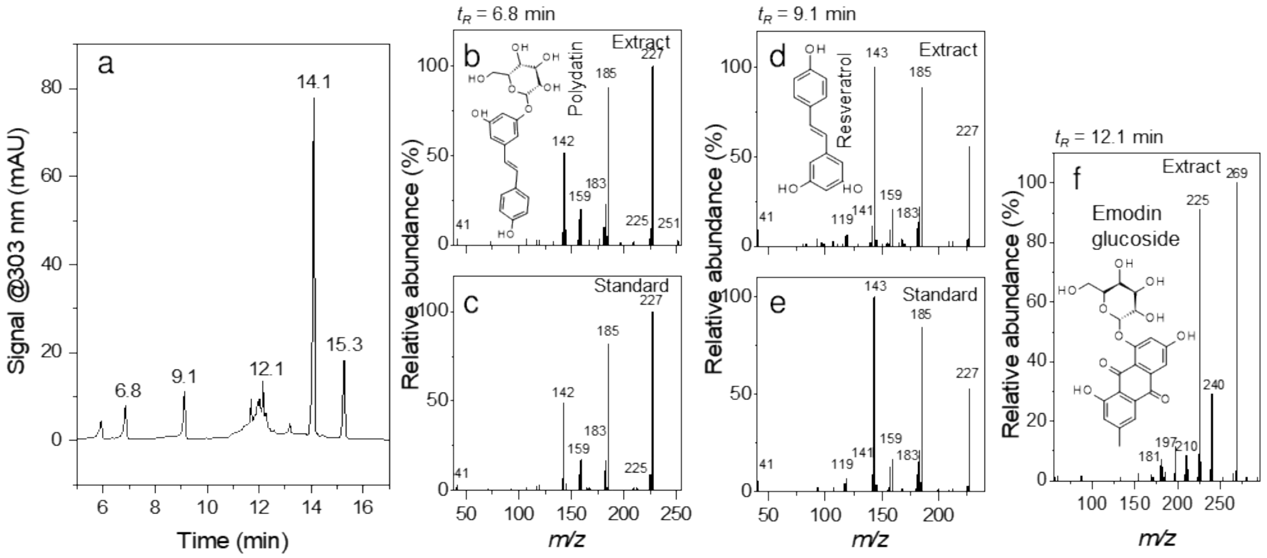

Figure S1. HPLC chromatogram of an ethanolic extract monitored at 303 nm (a). MS/MS spectra of the extracts and standards (b–f). Retention times of the eluted peaks are marked at their respective positions in the chromatogram.

The yields of synthesis as monitored by gravimetric analysis are shown in Table S1.

Table S1. Synthesis yields as measured by means of gravimetric analysis. The amount of Zn^{2+} was 1 mmol, hence the amount of base also gives the base : ZnAc ratio.

**n(LiOH×H_2O) (mmol)**	**η [%]**
0.5	26.8
1	53.3
1.5	83.3
2	110.4
2.5	121.6

3	111.7

The selected area electron diffraction (SAED) patterns together with simulated ones are shown in Fig. S2.

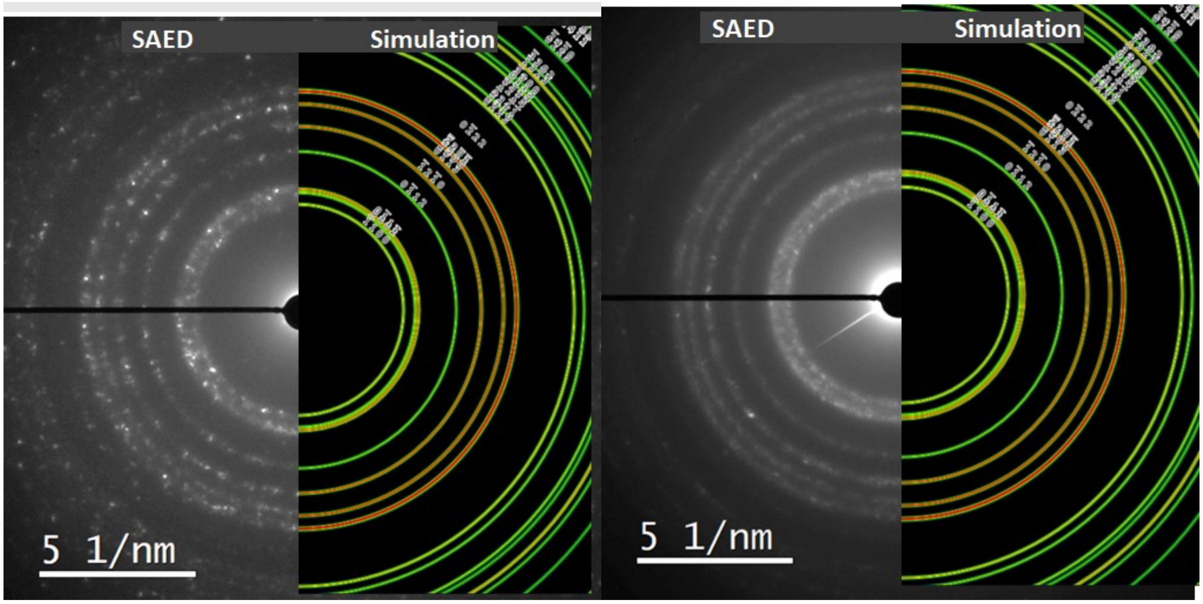

(a) (b)

Figure S2. Selected area electron diffraction (SAED) patterns of bare ZnO (a) and JK ZnO (b) obtained from TEM micrographs with the inset of the simulated ones.

Structural properties as obtained from the nitrogen isotherms at 77 K are shown in Table S2.

Table S2. Structural properties of the prepared photocatalysts.

	**Synthesis / annealing temperature (°C)**	**S_{BET} (m^2/g)**	**Pore size (nm)**	**Crystallite size (nm)**
Bare ZnO	80	68	22	10
JK ZnO	80	108	14	10

Bare ZnO	450	30	24	16
JK ZnO	450	41	20	14

The determination of the antimicrobial activity is shown in Fig. S3 below.

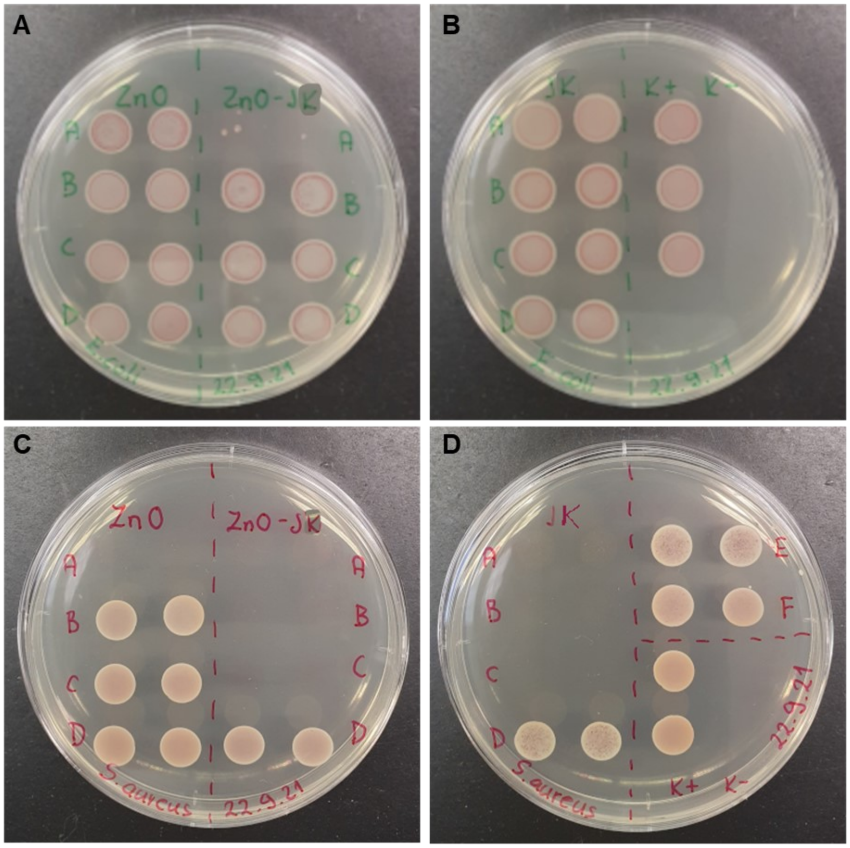

Figure S3. Results of testing the minimal bactericidal concentration against the two strains of bacteria; *E. coli* (top) and *S. aureus* (below). The letters in rows signify the concentration of ZnO NPs as follows: A: 2 mg/mL; B: 1 mg/mL; C: 0.5 mg/mL, D: 0.25 mg/mL.
